# Astragaloside IV Protects Against Oxidative Stress in Calf Small Intestine Epithelial Cells via NFE2L2-Antioxidant Response Element Signaling

**DOI:** 10.3390/ijms20246131

**Published:** 2019-12-05

**Authors:** Yafang Wang, Fugui Jiang, Haijian Cheng, Xiuwen Tan, Yifan Liu, Chen Wei, Enliang Song

**Affiliations:** 1 Institute of Animal Science and Veterinary Medicine, Shandong Academy of Agricultural Sciences, Sangyuan Road, Number 8, Jinan 250100, China; wangyafang6666@163.com (Y.W.); fgjiang2017@163.com (F.J.); 98061107@163.com (H.C.); weichenchen1989@126.com (C.W.); 2 Shandong Key Laboratory of Animal Disease Control and Breeding, Sangyuan Road, Number 8, Jinan 250100, China; 3 College of Life Sciences, Shandong Normal University, East Wenhua Road Number 88, Jinan 250014, China

**Keywords:** Astragaloside IV, small intestine epithelial cells, oxidative stress, hydrogen peroxide, nuclear factor erythroid 2-related factor 2, calf

## Abstract

Oxidative stress can damage intestinal epithelial cell integrity and function, causing gastrointestinal disorders. Astragaloside IV (ASIV) exhibits a variety of biological and pharmacological properties, including anti-inflammatory and antioxidant effects. The purpose of this research was to investigate the cytoprotective action of ASIV and its mechanisms in calf small intestine epithelial cells with hydrogen peroxide (H_2_O_2_)-induced oxidative stress. ASIV pretreatment not only increased cell survival, but it also decreased reactive oxygen species generation and apoptosis, enhanced superoxide dismutase, catalase, and glutathione peroxidase levels, and it reduced malondialdehyde formation. Furthermore, pretreatment with ASIV elevated the mRNA and protein levels of nuclear factor erythroid 2-related factor 2 (NFE2L2), heme oxygenase-1 (HMOX1), and NAD(P)H quinone dehydrogenase 1 (NQO1). The NFE2L2 inhibitor ML385 inhibited NFE2L2 expression and then blocked HMOX1 and NQO1 expression. These results demonstrate that ASIV treatment effectively protects against H_2_O_2_-induced oxidative damage in calf small intestine epithelial cells through the activation of the NFE2L2-antioxidant response element signaling pathway.

## 1. Introduction

The intestinal mucosa is one of the main points of contact between the body and the external environment [1]. Small intestinal epithelial cells are the first physiological line of defense in the mucosa, which selectively absorbs nutrients and protects against pathogenic invasions from the external environment [2]. Foreign pathogens and environmental changes can easily result in gastrointestinal diseases, such as infectious enterocolitis, irritable bowel syndrome, and inflammatory bowel disease [3]. Therefore, it is essential to maintain proper intestinal epithelial structure and function.

Oxidative stress, arising from the excessive generation of reactive oxygen species (ROS) and imbalances in the antioxidant system, can damage multiple cellular components through DNA hydroxylation, protein denaturation, lipid peroxidation, and membrane rupture. This outcome leads to apoptosis and other modes of cell death [4,5]. Given the small intestine of calf gastrointestinal hypoplasia, oxidative stress can disrupt the integrity and functioning of calf intestinal epithelial cells, thereby affecting absorption and allowing pathogen invasion and disease [6]. Oxidative stress plays a major role in the pathogenesis of a variety of disorders, including intestinal diseases [7,8,9,10]. Cellular defenses against ROS damage include both enzymatic and nonenzymatic antioxidant systems, which act to reestablish or maintain redox homeostasis [11]. Enzymatic antioxidant systems mainly involve enzymes that can scavenge or convert free radicals and ROS, such as superoxide dismutase (SOD), catalase (CAT), and glutathione peroxidase (GSH-Px), while nonenzymatic antioxidant systems involve synthetic and plant-extracted compounds [12]. Importantly, the activation of enzymatic antioxidant systems often requires an activated nonenzymatic antioxidant system as a precursor. Therefore, identifying safe and effective nonenzymatic antioxidants is essential to preventing and alleviating the damage caused by oxidative stress.

The nuclear factor erythroid 2-related factor 2 (NFE2L2)-antioxidant responsive element (ARE) signaling pathway plays a vital role in the cellular responses to oxidative stress-induced injuries [13]. NFE2L2 is a transcription factor that is highly sensitive to oxidative stress. It binds to AREs on the chromatin and promotes the transcription of a wide variety of antioxidant enzymes [14]. Normally, NFE2L2 is sequestered in the cytoplasm by Kelch-like ECH associated protein 1 (KEAP1). However, upon exposure to oxidative stress or chemopreventive compounds, NFE2L2 dissociates from KEAP1 and then translocate to the nucleus, where it heterodimerizes with its obligatory partner MAF bZIP transcription factor, binds AREs on the chromatin, and then activates ARE-mediated phase II detoxification by increasing the expression of a wide variety of antioxidant enzymes. This process includes heme oxygenase-1 (HMOX1), NAD(P)H quinone dehydrogenase 1 (NQO1), SOD, CAT, and GSH-Px [15,16].

**Figure 1:**
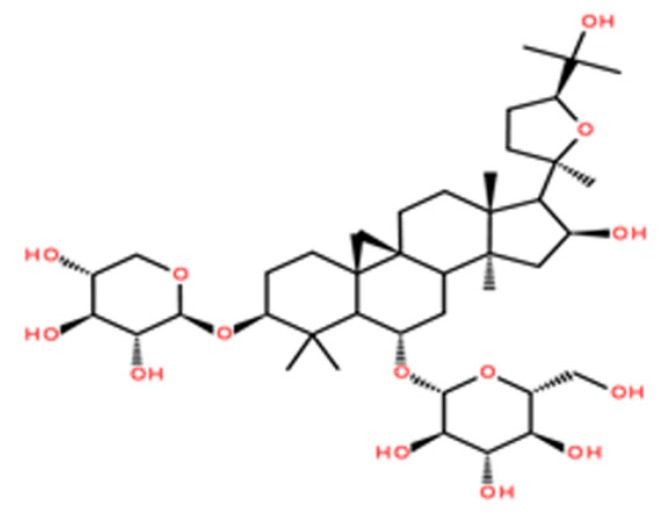
The chemical structure of astragaloside IV (ASIV).

Astragaloside IV (ASIV; Figure 1) is one of the main active components of the traditional Chinese medicinal plant *Astragalus membranaceus*. ASIV has several pharmacological properties, including anti-inflammatory, antiapoptotic, and antioxidative effects [17,18,19]. Recent studies have reported the strong antioxidative effects of ASIV, which can remove ROS and decrease lipid peroxidation [20,21]. However, the mechanisms by which ASIV ameliorates oxidative stress remain largely unknown. In this study, we investigated the protective role of ASIV against H_2_O_2_-induced oxidative stress in calf small intestine epithelial cells, as well as its mechanism of action.

## 2. Results

### 2.1. ASIV Inhibits H_2_O_2_-Induced Cell Cytotoxicity in Calf Small Intestine Epithelial Cells

**Figure 2:**
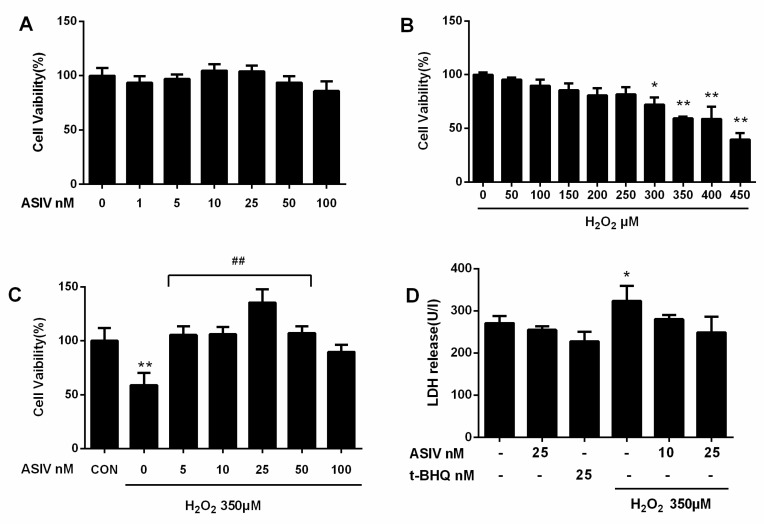
Effects of ASIV on H_2_O_2_-stimulated toxicity in calf small intestine epithelial cells. (**A**) Calf small intestine epithelial cells were treated with different concentrations of ASIV for 12 h, and cell viability was measured using the cell counting kit 8 (CCK8) assay; (**B**) Cells were exposed to different concentrations of H_2_O_2_ for 12 h, and cell viability was measured using the CCK8 assay; (**C**) Cells were pretreated with different concentrations of ASIV for 12 h before exposure to 350 μM H_2_O_2_ for 12 h, and cell viability was measured using the CCK8 assay; (**D**) Cells were pretreated with 10 or 25 nM ASIV or 25 nM tertiary butylhydroquinone (tBHQ) for 12 h before exposure to 350 μM H_2_O_2_ for 12 h, and lactate dehydrogenase (LDH) levels were assessed by an LDH release assay. Data represent the mean ± standard error of the mean (SEM). * *p* < 0.05 and ** *p* < 0.01 compared to control cells, ## *p* < 0.01 compared to H_2_O_2_-treated cells.

To investigate whether ASIV was cytotoxic to calf small intestine epithelial cells, we examined cell viability after treatment with 0–100 nM of ASIV for 12 h. As shown in Figure 2A, ASIV did not affect cell viability. Cells were also exposed to 0–450 μM H_2_O_2_ for 12 h, and viability decreased in a dose-dependent manner (Figure 2B). Compared to untreated cells, viability was significantly lower after treatment with 350 μM H_2_O_2_, which caused an approximately 59% loss in cell viability; where this concentration was used in subsequent experiments. Pretreatment with 5–100 nM ASIV for 12 h markedly attenuated the H_2_O_2_-induced decrease in cell viability (Figure 2C). Lactate dehydrogenase (LDH) levels in the culture medium of H_2_O_2_-treated cells were markedly increased compared to the untreated cells (Figure 2D), but they were not markedly different between the untreated cells and cells treated with ASIV (25 nM) or tertiary butylhydroquinone (tBHQ: 25 nM) without H_2_O_2_ treatment. Pretreatment with 25 nM ASIV (25 nM) for 12 h before H_2_O_2_ exposure reduced LDH release. These results indicated that ASIV exerted protective effects against H_2_O_2_-induced cytotoxicity.

### 2.2. ASIV Blocks H_2_O_2_-Induced Oxidative Stress Injury in Calf Small Intestine Epithelial Cells

**Figure 3:**
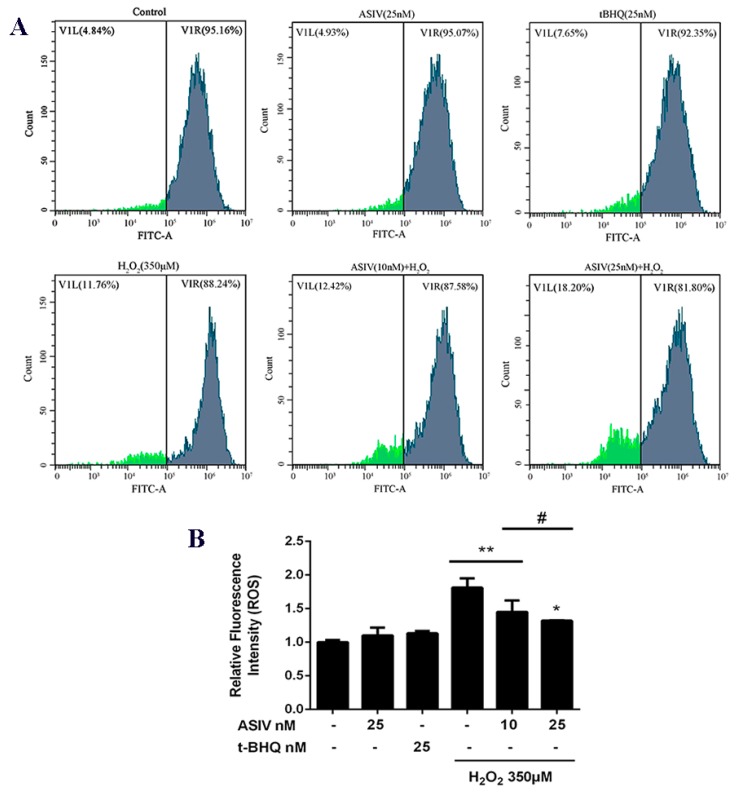
Effects of ASIV on H_2_O_2_-stimulated reactive oxygen species (ROS) generation in calf small intestine epithelial cells. (**A**) Cells were treated with or without ASIV (10 or 25 nM) or t-BHQ (25 nM) for 12 h before H_2_O_2_ (350 μM) exposure for 12 h and the ROS levels were detected by flow cytometry using 2′,7′-dichlorodihydrofluorescein diacetate (DCFH-DA). (**B**) Quantitative analysis of the mean DCF fluorescence intensity. Each value represents the mean ± SEM. * *p* < 0.05 and ** *p* < 0.01 compared to the control cells, # *p* < 0.05 compared to the H_2_O_2_-treated cells.

**Figure 4:**
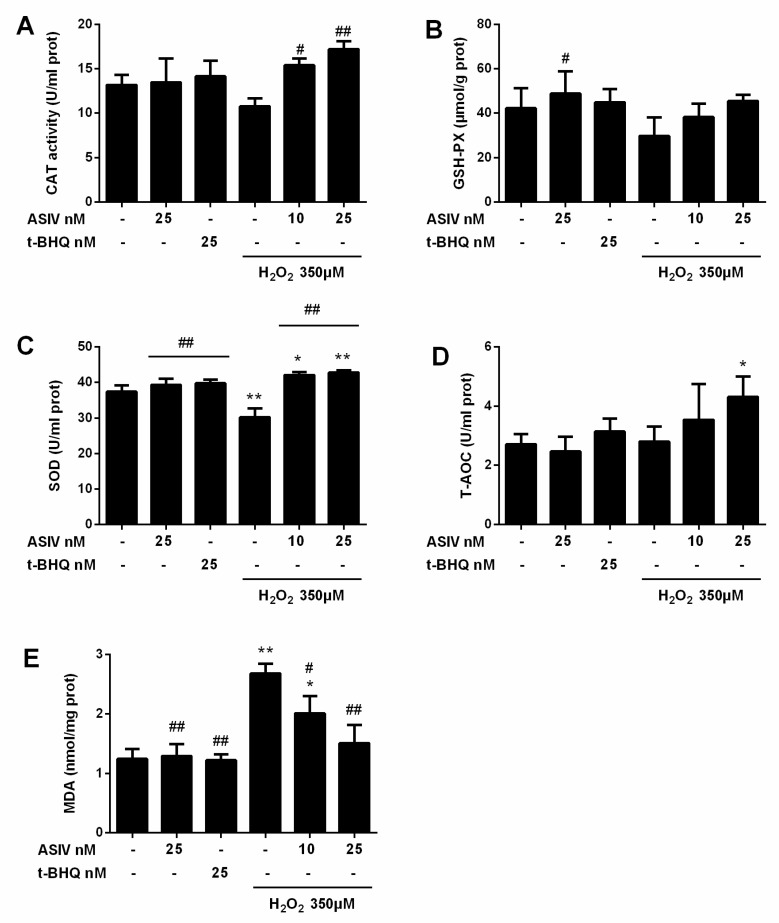
Effects of ASIV on catalase (CAT), glutathione peroxidase (GSH-Px), superoxide dismutase (SOD), total antioxidant capacity (T-AOC), and malondialdehyde (MDA) levels in H_2_O_2_-exposed calf small intestine epithelial cells. Cells were treated with or without ASIV (10 or 25 nM) and t-BHQ (25 nM) for 12 h, then subjected to 350 μM H_2_O_2_ for 12 h. (**A**) CAT activity, (**B**) GSH-Px content, (**C**) SOD activity, (**D**) T-AOC, and (**E**) MDA content were determined using commercial kits. Each value represents the mean ± SEM. * *p* < 0.05 and ** *p* < 0.01 compared to control cells, # *p* < 0.05 and ## *p* < 0.01 compared to H_2_O_2_-treated cells.

Next, we investigated whether ASIV protected against H_2_O_2_-stimulated oxidative damage in calf small intestine epithelial cells by reducing intracellular ROS generation. Intracellular ROS levels were increased by H_2_O_2_ stimulation, while ASIV pretreatment markedly inhibited intracellular ROS production in a dose-dependent manner (Figure 3). After exposure to H_2_O_2_ for 12 h, SOD levels were significantly reduced compared to the untreated cells, and CAT and GSH-Px levels were also decreased (Figure 4). When the cells were pretreated with 25 nM ASIV for 12 h before H_2_O_2_ exposure, ASIV enhanced the levels of CAT, GSH-Px, and SOD, as well as the total antioxidant capacity (T-AOC). H_2_O_2_ also significantly induced malondialdehyde (MDA) formation, which decreased with the ASIV pretreatment.

### 2.3. ASIV Decreases H2O2-Induced Apoptosis in Calf Small Intestine Epithelial Cells

**Figure 5:**
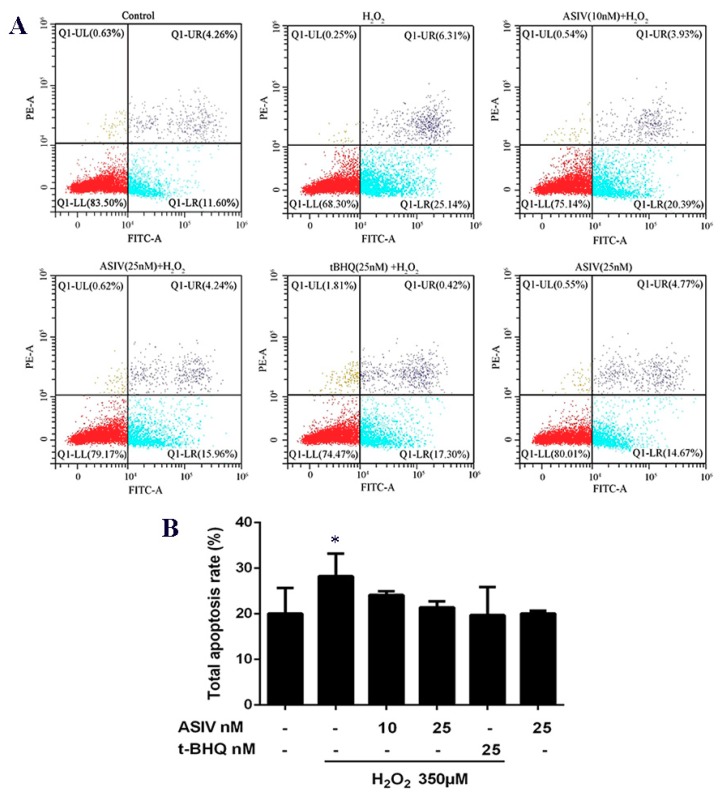
Effects of ASIV on H_2_O_2_-induced apoptosis in calf small intestine epithelial cells. (**A**) Cells were treated with or without ASIV (10 or 25 nM) and t-BHQ (25 nM) for 12 h, and then subjected to 350 μM H_2_O_2_ for 12 h. Apoptotic rates were detected using annexin V-fluorescein isothiocyanate (FITC) and propidium iodide (PI) staining and flow cytometry. (**B**) Quantitative analysis of total apoptosis rates. Each value represents the mean ± SEM. * *p* < 0.05 compared to control cells.

Calf small intestine epithelial cells used the annexin V-fluorescein isothiocyanate (FITC) and propidium iodide (PI), assayed by flow cytometry, to detect apoptosis. Stimulation with H_2_O_2_ caused an increase in apoptotic cells, which was reduced by ASIV pretreatment in a dose-dependent manner (Figure 5).

### 2.4. ASIV Activates NFE2L2-ARE Antioxidative Signaling in Calf Small Intestine Epithelial Cells

**Figure 6:**
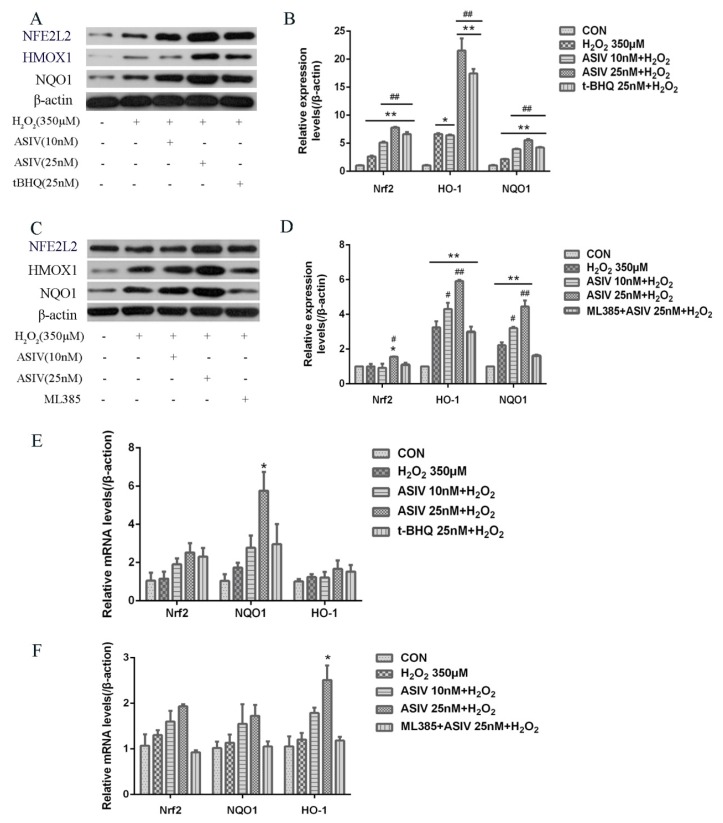
Effects of ASIV on the expression levels of nuclear factor erythroid 2-related factor 2 (NFE2L2), NAD(P)H quinone dehydrogenase 1 (NQO1), and heme oxygenase-1 (HMOX1) in H_2_O_2_-induced calf small intestine epithelial cells. (**A**) Cells were treated with or without ASIV (10 or 25 nM) and t-BHQ (25 nM) for 12 h and then were exposed to 350 μM H_2_O_2_ for 12 h. Relative protein levels were detected by western blotting; (**B**) Relative protein levels were determined by densitometric analysis; (**C**) Cells were treated with 5 μM ML385 for 24 h, ASIV (10 or 25 nM), and t-BHQ (25 nM) for the first 12 h, and 350 μM H_2_O_2_ for the last 12 h, and relative protein levels were detected by western blotting; (**D**) Relative protein levels were determined by densitometric analysis; (**E**,**F**) Relative mRNA expression levels of NFE2L2, NQO1, and HMOX1 were analyzed by qRT-PCR. Each value represents the mean ± SEM. * *p* < 0.05 and ** *p* < 0.01 compared to the control cells, # *p* < 0.05 and ## *p* < 0.01 compared to the H_2_O_2_-treated cells.

**Figure 7:**
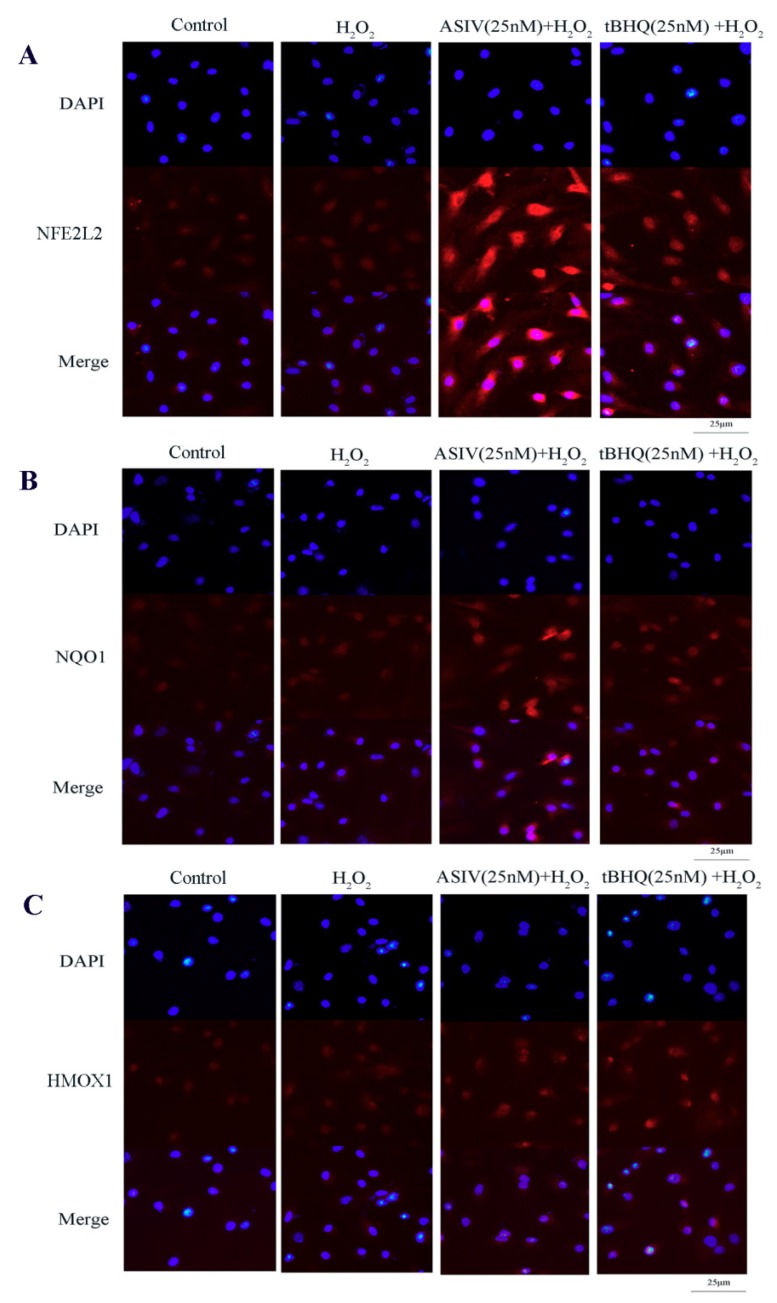
Effects of ASIV on nuclear NFE2L2, NQO1, and HMOX1 levels in calf small intestine epithelial cells. (**A**–**C**) Cells were treated with or without ASIV (10 or 25 nM) and t-BHQ (25 nM) for 12 h and then subjected to 350 μM H_2_O_2_ for 12 h. The distributions of NFE2L2, NQO1, and HMOX1 proteins were observed by immunofluorescent staining and confocal microscopy. Nuclear counterstaining was performed with 4′,6-diamidino-2-phenylindole (DAPI).

The NFE2L2-ARE signaling pathway plays a pivotal role in cellular defenses against oxidative stress. To examine whether this pathway is involved in the protective effects of ASIV against H_2_O_2_-induced oxidative stress, calf small intestine epithelial cells were pretreated with and without ASIV and t-BHQ before 12 h of H_2_O_2_ exposure. We examined the expression of NFE2L2/ARE-dependent antioxidant enzymes, including HMOX1 and NQO1, using the western blot and quantitative reverse transcription-polymerase chain reaction (qRT-PCR). Pretreatment with ASIV dose-dependently improved the protein levels of NFE2L2, HMOX1, and NQO1. With 25 μM ASIV, these proteins were significantly increased compared to their levels in the H_2_O_2_-treated cells (Figure 6A,B). Next, we used ML385, an NFE2L2 inhibitor, to confirm the involvement of NFE2L2-ARE signaling in the cytoprotective effects of ASIV. The inhibition of NFE2L2 by ML385 resulted in the significant downregulation of HMOX1 and NQO1 (Figure 6C,D). Furthermore, pretreatment with ASIV or t-BHQ led to dose-dependent and significant increases in the mRNA levels of NFE2L2, HMOX1, and NQO1 (Figure 6E), while ML385 inhibited this (Figure 6F). Enhanced NFE2L2, HMOX1, and NQO1 levels in the nucleus were confirmed by immunofluorescence and confocal microscopy (Figure 7).

Collectively, the results of this study provided evidence that ASIV activated the expression of NFE2L2, which promoted the transcription of the antioxidant enzymes HMOX1 and NQO1. Increased expression of these enzymes may protect calf small intestine epithelial cells from H_2_O_2_-induced oxidative stress and damage.

## 3. Discussion

The intestinal mucosa is one of the main barriers between the body and the external environment, and it functions by selectively absorbing nutrients and preventing invasion from the external environment. The integrity of the small intestine is essential for proper nutrient absorption, gut homeostasis, and organismal growth and health [6]. The production of ROS during oxidative stress creates an imbalance between the ability of intestinal epithelia to counteract to detoxify the harmful effects of ROS with intrinsic antioxidants and other protective mechanisms [22]. Furthermore, oxidative stress leads to immune system dysfunction and affects the digestion, absorption, and transformation of nutrients. Therefore, it is a critical factor in intestinal disease pathogenesis. ASIV, an active ingredient isolated from *Astragalus membranaceus*, has demonstrated antioxidative and protective effects on gastrointestinal health [23,24].

Numerous studies have demonstrated that H_2_O_2_ induces oxidative stress in various cell types, making it a classic in vitro model of oxidative stress [25,26,27]. ROS accumulation can cause severe cellular injury and result in decreased cell repair and regeneration [28]. In this study, we found that the viability of calf small intestine epithelia cells exposed to 350 μM H_2_O_2_ decreased by up to 41%, whereas ASIV pretreatment significantly inhibited H_2_O_2_-induced cytotoxicity and increased cell viability. Importantly, ASIV was not toxic to cells. In addition, ASIV pretreatment decreased H_2_O_2_-induced membrane damage and LDH release. These results indicated that ASIV had cytoprotective effects on calf intestine epithelia cells. Oxidative stress occurs when redox homeostasis is disrupted, and the cellular ROS overproduction results from the imbalance between oxidants and antioxidants [29]. SOD, GSH-Px, and CAT are key antioxidative enzymes in the defense against oxidative stress, and they act by scavenging ROS [30]. SOD and GSH-Px play an essential role in scavenging free radicals, and CAT can catalyze the decomposition of H_2_O_2_ to produce molecular oxygen (O_2_) and H_2_O [31]. Increased SOD, GSH-Px, and CAT levels contribute to the resolution of oxidative stress, and decreased levels exacerbate oxidative damage [22,32]. MDA levels also reflect the oxidative damage in calf small intestine epithelia cells, and it is crucial to protect cells by reducing ROS and MDA formation and enhance the SOD, GSH-Px, and CAT levels. In our study, the exposure of calf small intestine epithelia cells to H_2_O_2_ induced notable decreases in the SOD, GSH-Px, and CAT levels, in addition to marked increases in ROS and MDA production. However, ASIV pretreatment significantly reversed these effects, consistent with previous reports [33,34]. These results may have been because the accumulation of intracellular ROS caused protein denaturation, which led to glycosylation and decreased antioxidative enzyme activity [35]. ASIV can enhance the antioxidant capacity of cells by increasing the levels of antioxidant enzymes, thereby inhibiting cell damage caused by the accumulation of intracellular ROS. Oxidative stress usually induces apoptosis [36], and our results indicated that H_2_O_2_-induced apoptosis in calf small intestine epithelial cells was notably reduced by pretreatment with ASIV, reinforcing the notion that ASIV prevents H_2_O_2_-induced oxidative stress by increasing the endogenous antioxidant levels.

Activation of the antioxidant system is crucial to preventing oxidative damage. NFE2L2 is a target of exogenous toxic substances and oxidative stress, and it plays a vital role in the presence of these insults [37]. Normally, NFE2L2 binds to KEAP1 in the cytoplasm, which prevents its translocation to the nucleus and inhibits the transcription of a series of cytoprotective genes [38]. Under oxidative conditions, NFE2L2 dissociates from KEAP1 and translocates to the nucleus, where it binds to AREs and promotes the expression of numerous phase II enzymes, including HMOX1 and NQO1 [39]. Studies have demonstrated that the NFE2L2-ARE signal pathway could activate antioxidant enzymes, such as SOD, CAT, and GSH-Px, thereby enhancing the ability of small intestine epithelial cells to clean up ROS, protecting against oxidative damage [40] In this study, ASIV pretreatment increased the mRNA and protein expression levels of NFE2L2, HMOX1, and NQO1. To further investigate the role of NFE2L2-ARE signaling after H_2_O_2_ exposure, we used ML385, an inhibitor of NFE2L2, and found that ML385 markedly downregulated the expression levels of these proteins in the presence of ASIV. Moreover, the ASIV pretreatment enhanced the nuclear fluorescence intensity of NFE2L2, HMOX1, and NQO1. Collectively, these results indicate that the protective effects of ASIV against H_2_O_2_-induced calf small intestine epithelial cell oxidative damage may be attributed to the upregulation of HMOX1 and NQO1 via the NFE2L2-ARE signaling pathway, consistent with previous reports [22,41].

In conclusion, the present study indicates that ASIV treatment effectively protects against H_2_O_2_-induced oxidative damage, thereby inhibiting ROS overproduction, MDA formation, and apoptosis by upregulating SOD, GSH-Px, and CAT levels. The mechanisms underlying the protective effects of ASIV may be related to the activation of the NFE2L2-ARE signal pathway. More efforts are required to reveal the functions and mechanisms of ASIV on intestinal disease using further in vitro and in vivo studies.

## 4. Materials and Methods

### 4.1. Chemicals and Reagents

Astragaloside IV (> 98%) was purchased from Nanjing Spring & Autumn Biological Engineering (Nanjing, China) and stored in the dark at −20°C and then dissolved in dimethyl sulfoxide (DMSO) (Solarbio, Beijing, China). It was also diluted with complete medium prior to experimentation. H_2_O_2_ and t-BHQ were obtained from Sigma-Aldrich (St. Louis, MO, USA). Penicillin and streptomycin, Fetal bovine serum (FBS), and Dulbecco’s modified Eagle’s medium/nutrient mixture F-12 (DMEM/F-12) were obtained from Invitrogen-Gibco (Grand Island, NY, USA). Primary antibodies used targeted NFE2L2 (16396-1-AP, Proteintech, Chicago, IL, USA), NQO1 (11451-1-AP, Proteintech, Chicago, IL, USA), HMOX1 (ab13248, Abcam, Cambridge, UK), and β-actin (GB12001, Servicebio, Wuhan, China). GSH-Px, SOD, CAT, T-AOC, and MDA test kits were purchased from Nanjing Jiancheng Bioengineering Institute (Nanjing, China).

### 4.2. Cell Culture and Treatments

The Shandong Academy of Agricultural Sciences provided calf small intestine epithelial cells. Cells were cultured in DMEM/F-12 containing 10% FBS, 100 U/mL penicillin, and 100 U/mL streptomycin at 37 °C in a humidified atmosphere with 5% CO_2_. At 80% confluence, the cells were treated with 0, 10, or 25 nM ASIV for 12 h before treatment with 350 μM H_2_O_2_ for 12 h. Positive control cells were treated with 25 nM t-BHQ.

### 4.3. Cell Viability and LDH Leakage Assays

Cell viability was measured using the cell counting kit 8 (CCK8) assay (MedChemExpress, Monmouth Junction, NJ, USA) following the manufacturer’s instructions. Cells (2 × 10^4^ cells/well) were plated in 96-well plates and incubated for 24 h. Then they were incubated in H_2_O_2_ or ASIV for some time. The cells were then incubated in DMEM/F-12 containing 10% CCK8 reagent at 37 °C for 2 h. Absorbance at 450 nm was recorded with a microplate reader. LDH levels were measured by the Nanjing Jiancheng Bioengineering Institute (Nanjing, China).

### 4.4. Measurement of Intracellular ROS Levels

Intracellular ROS levels were evaluated using the ROS detection kit (Beyotime Institute of Biotechnology, Shanghai, China). Cells (1 × 10^6^ cells/mL) were cultured in 6-well plates, pretreated with different concentrations of ASIV or 25 nM t-BHQ for 12 h at 37 °C, and then they were treated with 350 μM H_2_O_2_ for 12 h at 37 °C. The cells were washed thrice with phosphate-buffered saline (PBS), incubated with 2′,7′-dichlorodihydrofluorescein diacetate (DCFH-DA) for 30 min, and then washed thrice with serum-free DMEM/F-12. Fluorescence intensities were measured by flow cytometry (BD Biosciences, Franklin Lakes, NJ, USA), with an excitation wavelength of 488 nm and an emission wavelength of 525 nm.

### 4.5. Apoptosis Analysis

Apoptosis used the Annexin V-FITC/PI apoptosis detection kit (Beyotime Institute of Biotechnology, Shanghai, China), and was determined by flow cytometry. The cells were washed twice with ice-cold PBS, digested and separated with 0.25% trypsin at 37 °C for 3 min, and then neutralized the trypsin with DMEM/F-12 containing 10% FBS. The cells were collected and centrifuged at 1500 rpm/min for 5 min. Next, the cells were resuspended in 100 μL binding buffer containing 5 μL Annexin V-FITC and 5 μL PI and then incubated at 4°C in the dark for 20 min. Apoptosis detection was performed by flow cytometry (BD Biosciences, Franklin Lakes, NJ, USA).

### 4.6. Measurement of the Intracellular GSH-Px, CAT, SOD, T-AOC, and MDA Levels

Cells (1 × 10^6^/mL) were plated in 6-well plates and then treated with ASIV or t-BHQ for 12 h before exposure to 350 μM H_2_O_2_ for 12 h. The GSH-Px, CAT, SOD, T-AOC, and MDA levels were quantified using commercially available kits, according to the manufacturer’s instructions.

### 4.7. RNA Extraction and qRT-PCR Analysis

Total RNA was isolated using TRIzol reagent (Invitrogen, Carlsbad, CA, USA) and then reverse transcribed into cDNA using the HiScript II Q RT SuperMix for qPCR (Vazyme, Nanjing, China), following the manufacturers’ instructions. The reverse transcription process was as follows: 50 °C for 15 min, and 85 °C for 5 s. The primer sequences are shown in Table 1, and they were synthesized by Sangon Biotech (Shanghai, China). We performed qRT-PCR on a LightCycler 480 (Roche Diagnostics, Burgess Hill, UK), using the ChamQ SYBR Color qPCR Master Mix (Vazyme, Nanjing, China) with the following conditions: stage 1, 95 °C for 30 s; stage 2, 40 cycles of 95 °C for 10 s and 60 °C for 30 s; and stage 3, 95 °C for 15 s, 60 °C for 60 s, and 95 °C for 15 s. Relative gene expression levels were determined using the 2^−ΔΔCT^ method [42,43].

### 4.8. NFE2L2, NQO1, and HMOX1 Immunofluorescence

Cells (1 × 10^6^/mL) were cultured in 24-well plates, rinsed thrice with PBS, and fixed with 4% paraformaldehyde for 30 min at room temperature. The cells were washed again with PBS, followed by permeabilization for 20 min in 0.1% Triton X-100. After washing with PBS, the cells were blocked in 5% FBS for 30 min. The blocking solution was removed and replaced with primary antibodies against NFE2L2 (1:200), NQO1 (1:50), and HMOX1 (1:300) in 0.5% FBS. The cells were then incubated overnight at 4 °C and then washed thrice with PBS before incubation with goat anti-rabbit (AS00029, GenStar, Beijing, China) or goat anti-mouse (AS00071, GenStar, Beijing, China) secondary antibodies for 1 h. The cells were washed thrice in PBS, and nuclear counterstaining was performed by incubation with 4′,6-diamidino-2-phenylindole (DAPI) for 10 min. Images were acquired using a BX51 fluorescence microscope (Olympus, Tokyo, Japan) and the Lecia Application Suite (Version 4.12.0, Germany) analysis software.

### 4.9. Western Blot Analysis

Cells were lysed in radioimmunoprecipitation buffer (G2002, Servicebio, Wuhan, China) containing protease and phosphatase inhibitors (G2007, Servicebio, Wuhan, China) for 30 min. Concentrations of protein were measured using a BCA protein assay kit (Beijing ComWin Biotech, Beijing, China). Equivalent amounts of proteins were resolved by 12% sodium dodecyl sulfate-polyacrylamide gel electrophoresis and then transferred to polyvinylidene difluoride membranes. The membrane was blocked with 5% (*w*/*v*) nonfat dry milk for 1 h and then incubation at 4 °C overnight with primary antibodies against NFE2L2 (1:1000), NQO1 (1:500), HMOX1 (1:250), and β-actin (1:1500). After washing thrice with 0.5% Tris-buffered saline/TWEEN (TBST), the membrane were incubated with horseradish peroxidase-conjugated goat anti-rabbit (GB23303, Servicebio, Wuhan, China) or goat anti-mouse antibodies (GB23301, Servicebio, Wuhan, China) for 2 h at room temperature. The bands were detected by an ECL (G2014, Servicebio, Wuhan, China), and the band intensities were quantified using the Alpha analysis software (alphaEaseFC, Alpha Innotech, Santa Clara, CA, USA).

### 4.10. Statistical Analysis

All data are presented as the mean ± standard error of the mean (SEM). Comparisons between different groups were analyzed using a one-way analysis of variance and Tukey′s post hoc test in SAS V8 (SAS Institute, Cary, NC, USA). Statistical significance was defined as *p* < 0.05 or *p* < 0.01.

## Figures and Tables

**Table 1:** Primer for quantitative real-time PCR.

Primer Name	Primer Sequence (5′-3′)	Product Length (bp)
NFE2L2	Sense Primer: ACCCAGTCCAACCTTTGTCGT	143
	Anti-sence Primer: GCGGCTTGAATGTTTGTCTTT	
NQO1	Sense Primer: CGGCTCCATGTACTCTCTGC	183
	Anti-sence Primer: TCCAGGCGTTTCTTCCATCC	
HMOX1	Sense Primer: CAAGCGCTATGTTCAGCGAC	198
	Anti-sence Primer: TTGGTGGCACTGGCGATATT	
β-actin	Sense Primer: CACCGCAAATGCTTCTAGGC	186
	Anti-sence Primer: TGTCACCTTCACCGTTCCAG	

NFE2L2, NF-E2-related factor 2; NQO1, NAD(P)H quinone dehydrogenase 1; HMOX1, heme oxygenase-1.

## Abbreviations

ARE	antioxidant responsive element
ASIV	Astragaloside IV
CAT	Catalase
CCK8	Cell counting kit 8
DAPI	4′,6-diamidino-2-phenylindole
DCFH-DA	2′,7′-dichlorodihydrofluorescein diacetate
NFE2L2	nuclear factor erythroid 2-related factor 2
NQO1	NAD(P)H quinone dehydrogenase 1
O_2_	molecular oxygen
qRT-PCR	quantitative reverse transcription-polymerase chain reaction
ROS	reactive oxygen species
SOD	superoxide dismutase
T-AOC	total antioxidant capacity
tBHQ	tertiary butylhydroquinone
